# Long‐Term Outcomes of Catheter Ablation in Ventricular Tachycardia Electrical Storm: A Retrospective Cohort Study

**DOI:** 10.1002/clc.70221

**Published:** 2025-11-15

**Authors:** Cem Çöteli, Samuray Zekeriyayev, Can Sezer, Hikmet Yorgun, Kudret Aytemir

**Affiliations:** ^1^ Department of Cardiology Hacettepe University Adult Hospital Altındağ Ankara Türkiye; ^2^ Department of Cardiology, Cardiovascular Research Institute Maastricht (CARIM), Maastricht University Medical Center Maastricht the Netherlands

## Abstract

**Background and Objective:**

Electrical storm is a life‐threatening condition commonly observed in patients with structural heart disease. While catheter ablation has emerged as an effective treatment for electrical storm, the long‐term outcomes are still unknown. This study aims to evaluate the long‐term outcomes of catheter ablation in patients with electrical storm, focusing on mortality, ventricular tachycardia (VT) recurrence, and hospitalization rates.

**Methods:**

We conducted a retrospective cohort study at a single center, enrolling 65 patients admitted with electrical storm. All patients underwent catheter ablation The primary outcome was VT‐related ICD therapies, while the secondary outcomes included all caused mortality, VT‐related ICD therapies, repeat ablation, hospitalization, and stroke.

**Results:**

The cohort was predominantly male (86.15%) with ischemic cardiomyopathy (56.92%) and a mean left ventricular ejection fraction (LVEF) of 35.3% ± 13%. All procedures were completed without any fatalities and without significant complications in 93.85% of cases. During follow‐up, 22 patients (33.85%) received ICD therapies for VT. The median estimated survival time for the VT‐free survival was 43 months. The 12‐month mortality rate was 26.15%. Over the median follow‐up of 23 months, 40% of patients died, and 72% experienced a composite endpoint of death, VT recurrence, or hospitalization. Multivariate analysis identified reduced LVEF as the strongest predictor of mortality during follow‐up.

**Conclusion:**

VT ablation is a safe and effective therapeutic option for managing electrical storm, providing high acute procedural success and allowing most patients to be discharged. However, this high‐risk population remains at significant risk for long‐term morbidity and mortality.

## Graphical abstract

Despite high acute success, catheter ablation for electrical storm is associated with substantial recurrence and mortality. This study emphasizes the complex substrate and high‐risk profile of this population, underscoring the need for careful long‐term management.

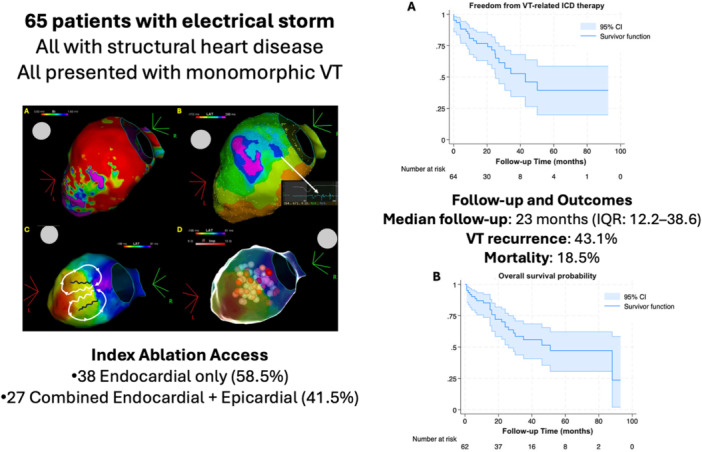

## 1 Introduction

Ventricular tachycardia (VT) is a severe arrhythmia, particularly prevalent in patients with structural heart disease and heart failure [1, 2]. Electrical storm (ES) is a severe form of ventricular arrhythmia, which is characterized by ≥ 3 VT episodes occurring within 24 h [3]. As this condition constitutes a critical emergency leading to hemodynamic instability and resistance to standard antiarrhythmic treatments, optimal management of the condition is paramount to increase survival. Addressing ESs typically requires immediate and multifaceted interventions.

Although antiarrhythmic drugs (AAD) and implantable cardioverter defibrillators (ICDs) are essential in managing (VT), catheter ablation has surfaced as an increasingly viable treatment, particularly for patients experiencing ES [4, 5]. Catheter ablation not only decreases the immediate VT burden effectively, but also provides a chance for lasting rhythm stabilization [3]. Nonetheless, long‐term results of catheter ablation remain inadequately understood, especially for patients hospitalized due to ES.

This study aims to address this gap by assessing the long‐term impact of catheter ablation of VT in patients presenting with ES.

## 2 Methods

### 2.1 Study Design and Study Population

This retrospective cohort study was conducted at the Hacettepe University Cardiology Department from November 2014 to May 2022. It involved patients admitted due to ES, defined as having ≥3 episodes of sustained VT lasting 30 s or more, or receiving appropriate ICD therapy for VT within 24 h, as noted in ICD records logs. Patients with ventricular fibrillation (VF) or polymorphic VT (including torsades de pointes) as the primary arrhythmia in ICD records were excluded. Only patients with sustained monomorphic VT episodes or monomorphic VT that triggered ICD therapies were included.

- Patients were eligible for inclusion if they were 18 years or older, had a documented ES upon admission, and underwent catheter ablation as part of their treatment. The criteria for exclusion included:
- Individuals who underwent cardiopulmonary arrest before the VT ablation procedure,
- Patients receiving recurrent shock therapy for VF or polymorphic VT,
- This retrospective study was approved by the Hacettepe University Health Sciences Research Ethics Committee.

### 2.2 Data Collection

Patient data were collected retrospectively from the hospital′s electronic medical record system. Variables included patient demographics (age, sex, and comorbidities), clinical history (underlying cardiac conditions, prior VT episodes), procedural details (ablation approach and complications), and postprocedural outcomes (VT recurrence, hospital readmissions, mortality. Device interrogation logs were reviewed to evaluate VT episodes and appropriate ICD therapies for patients with ICDs, including ICD shocks, anti‐tachycardia pacing (ATP), and both sustained and non‐sustained VT episodes occurring within the last 6 months before ablation and during post‐procedural follow‐up.

### 2.3 VT Ablation Procedure

#### 2.3.1 Preprocedural Planning

Patients received a comprehensive evaluation that included a 12‐lead electrocardiogram (ECG), transthoracic echocardiography, and routine blood analysis (complete blood count, serum biochemistry, and electrolytes). Any identified serum electrolyte imbalances were promptly corrected to enhance arrhythmia management and minimize procedural risks risk.

For patients with no contraindications, intravenous amiodarone was given to stabilize the arrhythmia. For patients who could tolerate beta‐blockers, intravenous esmolol was administered while continuously monitoring invasive blood pressure. If stabilization failed despite these medications, patients were sedated and intubated to ensure hemodynamic stability support.

In stabilized patients, cardiac imaging before the procedure, including cardiac magnetic resonance imaging (MRI) or computed tomography (CT), was performed to assess structural abnormalities, the burden, and the location of myocardial scars. Transesophageal echocardiography (TEE) was performed in patients with atrial fibrillation (AF) to rule out thrombus formation in the left atrium and left atrial appendage.

For patients who remained hemodynamically unstable despite AADs and sedation, VT ablation was planned within 24 h of hospitalization to prevent further clinical deterioration and manage life‐threatening arrhythmias.

#### 2.3.2 Ablation Procedure

All procedures were conducted under general anesthesia or deep sedation and local anesthesia with continuous invasive blood pressure monitoring. The endo‐epicardial approach was planned for patients with non‐ischemic cardiomyopathy (NICMP) or evidence of epicardial scarring identified on preprocedural cardiac MRI, provided there was no history of prior cardiac surgery. The epicardial puncture was achieved using the subxiphoid approach utilizing the Sosa technique [6]. After the epicardial puncture, Agilis NxT Steerable Introducer was positioned in the epicardial space before systemic heparinization. Retro aortic and transseptal access was planned for patients without mechanical aortic or mitral valves. The transseptal puncture was performed using the Brockenbrough needle, followed by placing Agilis NxT Steerable Introducer in the left atrium before heparin administration. Following epicardial and transseptal access, 80 U/kg intravenous heparin was administered as a bolus. Activated clotting time (ACT) was monitored every 15 min and maintained between 300 and 500 ms.

**Figure 1:**
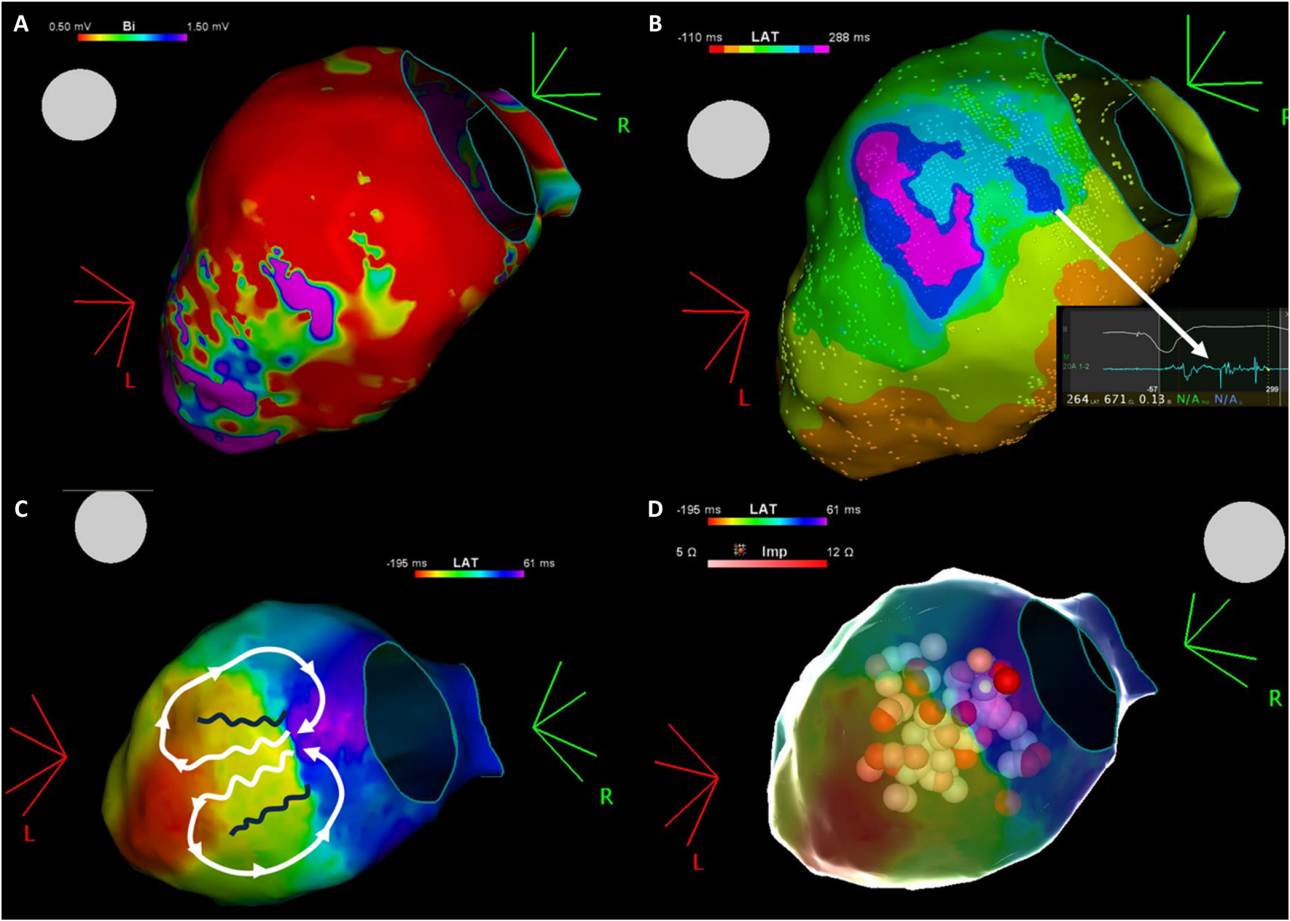
(A) Voltage map of the left ventricle. (B) Isochronal late activation map under right ventricular pacing, with the white arrow indicating a fragmented late potential signal in the deceleration zone. (C) Activation map during clinical VT, where the critical isthmus, of intramural origin, is located between the navy lines, and white arrows highlight the endocardial figure‐of‐eight propagation pattern of VT. (D) Ablation area targeting the critical isthmus.

All procedures utilized 3D electroanatomical mapping systems, EnSite Precision (Abbott), EnSite X EP System (Abbott), or CARTO 3 System (Johnson & Johnson). If obtained, voltage (Figure 1a) and late potential mapping (Figure 1b) of the left ventricular endocardium and epicardium were performed during sinus rhythm or right ventricular pacing, utilizing multipolar high‐density mapping catheters, Advisor HD Grid Mapping Catheter (Abbott), Optima Mapping Catheter (Abbott), PENTARAY NAV ECO High‐Density Mapping Catheter (Johnson & Johnson) or DECANAV Mapping Catheter (Johnson & Johnson). Late potential mapping was performed to identify deceleration zones. Late potentials and LAVAs were annotated during mapping. Decremental Evoked Potential (DEEP) mapping was performed in patients who could tolerate ventricular pacing after 2021 [7]. VT was induced through programmed and burst ventricular pacing. If VT was induced, its circuit—including entrances, isthmuses, and exits—was mapped using activation mapping (Figure 1d) techniques.

The ablation zone was chosen to target tachycardia isthmuses and late potentials (Figure 1d), utilizing irrigated RF ablation catheters such as Flexability (Abbott), TactiCath Contact Force Ablation Catheter(Abbott), THERMOCOOL Catheter (Johnson & Johnson), or THERMOCOOL SMARTTOUCH Catheter (Johnson & Johnson). After ablation, VT induction was repeated through programmed stimulation up to 3 extrastimuli and burst pacing to verify non‐inducibility. If stable VT induction occurred, activation mapping was conducted again, and the ablation zone was extended to include additional critical area isthmuses.

The procedural success was defined as the termination of clinical VT and non‐inducibility of VT in patients with sustained and inducible VT. If there was no inducible VT, procedural success was defined as the elimination of LPs and LAVAs, including deceleration zones of ILAM if obtained. To assess and manage potential active ischemia, all patients with ischemic cardiomyopathy (ICMP) underwent diagnostic coronary angiography following the ablation procedure.

### 2.4 Postprocedural Follow‐Up

All patients were monitored in the intensive care unit (ICU) for at least 24−48 h post‐procedure. They were extubated within 24 h after the procedure, provided they achieved hemodynamic stability and adequate respiratory function. A pigtail catheter was used for continuous drainage in patients with epicardial access and removed after ensuring no significant pericardial effusion. Before removal, intrapericardial triamcinolone (1 mg/kg) was administered to reduce inflammation.

Postprocedural heart failure therapy was tailored to each patient, considering their baseline functional status and any new procedural findings. AAD treatment was individualized based on each patient's arrhythmic burden, comorbidities, and contraindications. Patients at high risk of recurrent VT were maintained on amiodarone unless contraindicated.

### 2.5 Study Outcomes

The primary outcome of the study was the VT‐related ICD therapies, which included shock and ATP therapies. VT‐related ICD therapies were reviewed using hospital records. Mortality data were obtained from hospital and national death records registries. Secondary outcomes included:

- All‐cause mortality.
- VT‐related ICD therapies: Defined as appropriate ICD interventions for VT, including ATP or shock therapy.
- Redo procedure for VT: Recurrent VT ablation during the follow‐up.
- Hospitalizations: Defined as unplanned readmissions for VT, heart failure, or other cardiac‐related events within the follow‐up period.
- Stroke: Defined as any new ischemic or hemorrhagic stroke documented in medical records during the follow‐up period.

### 2.6 Long‐Term Follow‐Up

Outpatient follow‐up visits were scheduled at 1 month, 6 months, and then every 6 months thereafter. Patients underwent clinical evaluations, ICD interrogations, and imaging and laboratory assessments during these visits as required. Data collected during follow‐up included the frequency of VT episodes, medication adjustments, and any postprocedural complications or hospitalizations.

Phone calls were conducted to obtain missing information for patients whose data were incomplete in the hospital′s electronic records. Patients or their caregivers were contacted to confirm key outcomes, such as recurrence of VT, changes in medication, and hospitalization, which were used during phone calls to ensure consistency in data collection.

### 2.7 Statistical Analysis

Statistical analyses were performed using Stata 18 software. Continuous variables were tested for normality using the Shapiro−Wilk test. Data with a normal distribution were presented as mean ± standard deviation (SD), while non‐normally distributed data were summarized as median with minimum and maximum values.

The primary and secondary outcomes were analyzed using Kaplan−Meier survival curves to evaluate event‐free survival.

Univariate and multivariate analyses were performed using Cox proportional hazards regression models to identify mortality‐related variables. Variables with a *p* < 0.05 in univariate analysis were included in the multivariate analysis to control for potential confounders. Hazard ratios (HR) with 95% confidence intervals (CI) were reported to quantify the strength of associations.

## 3 Results

### 3.1 Baseline Characteristics

The study population consisted of 65 patients (86.1% male, median age: 65 (18–85). The primary underlying cardiomyopathy types included ICMP in 37 patients (56.9%), idiopathic NICMP in 26.15%, ARVD in 8 (12.3%) and HCMP in 3 (4.6%).

Heart failure was highly prevalent, affecting 87.7% of patients, with a mean left ventricular ejection fraction (LVEF) of 35.3 ± 13%. AF was present in 29.23% of the study population.

Fifty‐five patients (84.6%) had implantable cardioverter‐defibrillators (ICDs) before ablation. Among these, ICD interrogation revealed that nine patients had documented episodes of non‐sustained VT and/or sustained VT requiring ICD therapy, and five patients had received ICD shocks and/or ATP therapies within the past 6 months.

Eighteen patients (27.7%) had a history of previous VT ablation. Among them, four patients had undergone two prior ablation procedures, and two patients had undergone three.

Before ablation, 48 patients (73.8%) were on AAD therapy. Amiodarone was the most commonly used AAD (38 patients), followed by propafenone (3 patients), mexiletine (3 patients), flecainide (2 patients), and a combination of amiodarone and mexiletine (2 patients). Seventeen patients (26.2%) were not on any AADs before ablation.

Baseline characteristics are summarized in Table 1.

**Table 1:** Baseline characteristics of the study population.

	(*n* = 65)
Age, median (min−max)	65 (18–85)
Male Gender, *n* (%)	56 (86.2%)
Cardiomyopathy type	
ICMP, *n* (%)	37 (56.9%)
NICMP, *n* (%)	17 (26.2%)
ARVD, *n* (%)	8 (12.31%)
HCMP, *n* (%)	3 (4.6%)
Hypertension, *n* (%)	41 (63.1%)
Diabetes mellitus, *n* (%)	15 (23.1%)
Heart failure with reduced EF, *n* (%)	57 (87.7%)
ICD	55 (84.6%)
CAD, *n* (%)	38 (58.5%)
AF, *n* (%)	19 (29.2%)
CVE, *n* (%)	6 (9.2%)
Previous VT ablation history, *n* (%)	18 (27.7%)
LVEF (%), mean ± SD	35.3 ± 13
LVEDD (mm), mean ± SD	58.9 ± 10.5
Severe mitral regurgitation	7 (10.8%)
Metallic mitral prosthesis	1 (1.5%)
Metallic aortic prosthesis	1 (1.5%)
Hemoglobin (g/dL), mean ± SD	13.3 ± 2.1
Glomerular filtration rate (mL/min), mean ± SD	72.7 ± 25.6
BNP, median (min−max)	433 (12.3−4583)
Use of AAD before ablation—*n* (%)	48 (73.8%)
Amiodarone, *n*	38
Propafenone, *n*	3
Mexiletine, *n*	3
Flecainide, *n*	2
Amiodarone + Mexiletine, *n*	2

Abbreviations: AAD, antiarrhythmic drugs; AF, atrial fibrillation; ARVD, arrhythmogenic right ventricular dysplasia; BNP, brain natriuretic peptide; CAD, coronary artery disease; CVE, cerebrovascular event; HCMP, hypertrophic cardiomyopathy; ICD, implantable cardioverter defibrillator; ICMP, ischemic cardiomyopathy; LVEDD, left ventricle end diastolic diameter; LVEF, left ventricular ejection fraction; NICMP, non‐ischemic cardiomyopathy; VT, ventricular tachycardia.

### 3.2 Procedural Findings

In 22 (33.9%) patients, VT ablation was performed within the first 24 h following hospital admission due to electrical and hemodynamic instability despite the use of AADs and sedation. In the remaining 43 patients, VT ablation was performed within a median of 4 (2−12) days following admission.

The average procedure duration was 193 ± 54 min, with an average fluoroscopy time of 25 ± 5.5 min. No patients required mechanical circulatory support (e.g., intra‐aortic balloon pump, Impella, or ECMO) during the ablation.

A total of 37 procedures (56.9%) were conducted using the CARTO 3 System (Johnson & Johnson), 26 (40.0%) with the EnSite Precision (Abbott), and 2 (3.1%) using the EnSite X EP System (Abbott).

Ventricular access was via endocardial approach in 38 patients (58.46%), while a combined endo‐epicardial access was performed in 27 patients (41.54%).

**Figure 2:**
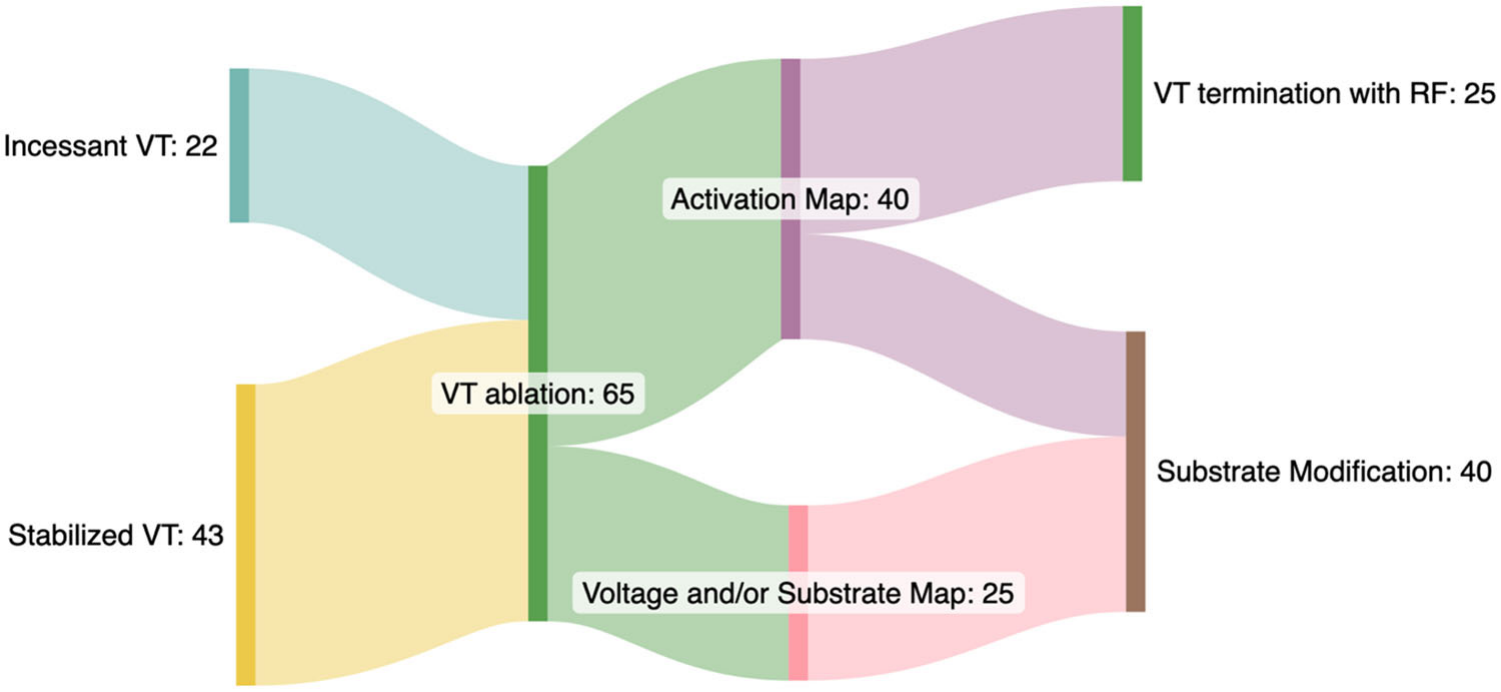
VT ablation procedures of the patients.

Activation mapping was conducted in 40 patients (61.54%). Among these, the median number of VT morphologies was 2 (1−6). In 25 (38.9%) patients, clinical VT was successfully terminated with RF ablation, and VT isthmus ablation was achieved. In 40 patients, substrate modification was performed, including scar homogenization and LPs and LAVAs ablation (Figure 2).

In 57 patients, LPs and LAVAs were annotated during the ablation, and following the ablation, the elimination of these signals was monitored. Ablation was performed endocardially in 41 patients (63.08%), using an epicardial approach in 5 patients (7.69%), and a combined endo‐epicardial approach in 19 patients (29.23%). All 65 patients (100%) achieved acute procedural success, non‐inducibility of clinical VT, or elimination of LPs and LAVAs. The critical isthmuses of clinical VTs and the ablation zone are detailed in Table 2.

**Table 2:** Procedural findings.

	*n* (%)
Emergent VT ablation	22 (33.9%)
Anesthesia	
General anesthesia	37 (56.9%)
Deep sedation + local anesthesia	28 (43.1%)
Access	
Endocardial	38 (58.5%)
Endo + epicardial	27 (41.5%)
Mean fluoroscopy time, min	25 ± 5.5
Mean procedure duration, min	193 ± 54
3D Ablation System	
CARTO 3	37 (56.9%)
Mapping catheter	
PENTARAY NAV ECO mapping catheter	23 (62.2%)
◦ DECANAV mapping catheter	14 (37.8%)
◦ Ablation catheter	
THERMOCOOL catheter	30 (81.1%)
◦ THERMOCOOL SMARTTOUCH catheter	7 (18.9%)
◦ EnSite precision	26 (40.0%)
EnSite X	2 (3.1%)
Mapping catheter	
Advisor HD grid	19 (67.9%)
◦ Optima	9 (32.1%)
◦ Ablation catheter	
TactiCath contact force ablation catheter	18 (64.3%)
FlexAbility ablation catheter	10 (35.7%)
Activation map	40 (61.5%)
Number of VT morphology	2 (1–6)
Termination with RF	25 (38.7%)
TCL of VTs	334.4 ± 46.5
Ablation location	
Endocardial	41 (63.1%)
Epicardial	5 (7.7%)
Endo + epicardial	19 (29.23%)
IDCMP	37
Inferolateral	10 (27%)
Inferior	9 (24.3%)
Inferoseptal	5 (13.5%)
Septal	5 (13.5%)
Anteroapical	3 (8.1%)
Lateral	3 (8.1%)
Anterolateral	1 (2.7%)
AL pap musc	1 (2.7%)
NIDCMP	17
Inferolateral	5 (29.4%)
Inferior	4 (23.5%)
Septal	6 (35.3%)
Anterolateral	2 (11.8%)
ARVD	8
RV anterolateral	7 (87.5%)
RV anteroseptal	1 (14.3%)
HCMP	3
Apical aneurysm	3 (100%)

One patient underwent percutaneous coronary intervention (PCI) before discharge due to a significant and revascularizable lesion. In another patient, although a hemodynamically significant coronary stenosis was identified, the coronary anatomy was not suitable for PCI; therefore, medical management was pursued. Non‐invasive ischemia testing (e.g., myocardial perfusion scintigraphy, stress echocardiography, or treadmill testing) was not performed in any patient.

Periprocedural complications were reported in seven patients (10.8%). Intrahospital mortality after ablation occurred in three patients (4.62%). Vascular access site complications included hematoma (3, 4.6%) and arteriovenous fistula (1, 1.5%) observed in 3 patients (4.6%), pneumonia and septicemia in 2 patients (3.08%), and pulmonary thromboembolism (PTE) in 1 patient (1.5%).

### 3.3 Follow‐Up Outcomes

After the procedure, 49 patients (75.4%) were discharged on AAD therapy. Amiodarone was the most commonly prescribed drug (*n* = 38), followed by sotalol (*n* = 6), mexiletine (*n* = 3), and propafenone (*n* = 1). One patient was treated with a combination of amiodarone and sotalol. Sixteen patients (24.6%) did not receive AAD therapy after ablation.

The median duration of hospitalization was 7 days, ranging from 2 to 51 days. Three patients (4.6%) died during the hospitalization period. These in‐hospital deaths were not related to procedural complications but occurred in the context of progressive cardiogenic shock and multi‐organ failure during the postprocedural recovery phase.

The median follow‐up duration was 23 months (IQR: 12.2–38.6 months). Three patients (4.62%) died before discharge, while 26 patients (40.0%) deceased during follow‐up, (19 [29.2%] fatalities related to cardiac disorders and 7 [10.8%] due to non‐cardiac causes). The median estimated survival time for the primary outcome was 51 months.

During follow‐up, 22 patients (33.9%) received appropriate ICD therapies. Additionally, 7 patients had non‐sustained VT episodes detected during routine ICD checks, but they did not require therapy. The median estimated survival time without sustained VT was 43 months.

A repeat ablation procedure was performed during follow‐up in 18 patients (27.7%). All of these patients underwent endocardial mapping and ablation in the redo procedure. In 8 patients (44.4%), additional epicardial ablation was required during the redo procedure. Notably, two of these patients had not undergone epicardial ablation during their index procedure. The anatomical targets during redo ablation were either the same as or adjacent to the ablation sites of the index procedure.

During the follow‐up, 47 patients (72.3%) experienced secondary outcomes, including hospitalization, ICD therapy, repeat VT ablation, and stroke. Of these, 40 patients (61.54%) were hospitalized due to cardiac issues, such as heart failure decompensation and ventricular arrhythmias. The median estimated survival time free of secondary outcomes was 26 months.

Routine postprocedural anticoagulation was not administered. Anticoagulation therapy was continued only in patients with specific indications, such as AF or mechanical heart valves.

**Figure 3:**
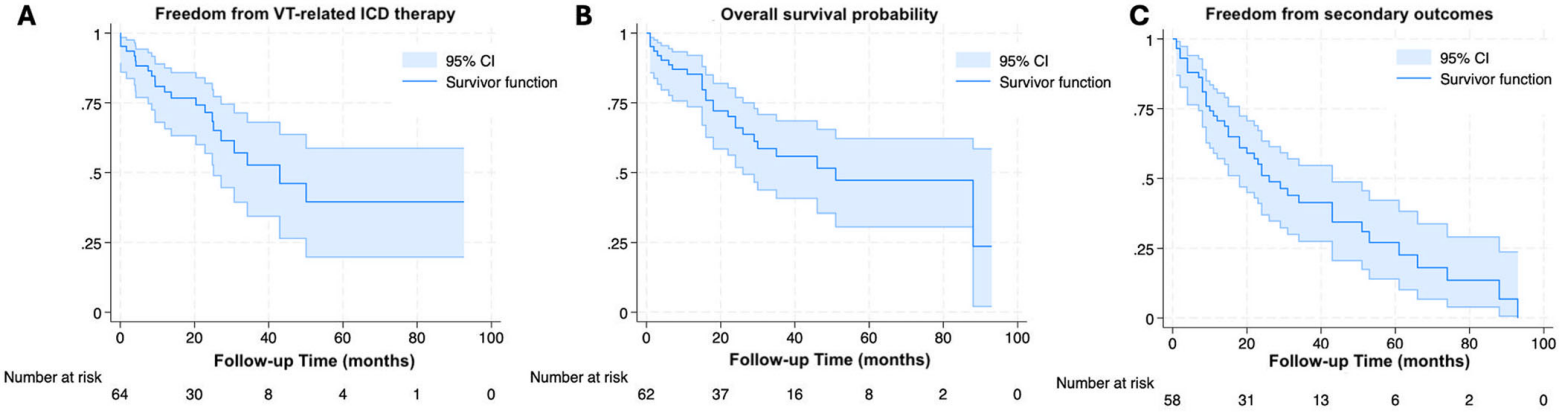
Kaplan–Meier survival curves illustrating clinical outcomes during follow‐up. (A) Freedom from VT‐related ICD therapies. (B) Overall survival. (C) Freedom from composite secondary outcomes (all‐cause mortality, VT recurrence, hospitalization, and stroke). *Time is expressed in months; y‐axis represents survival probability*.

Detailed data on ablation access route, ablation location (endo‐/epicardial), and redo procedures are summarized in Table 3, and Kaplan−Meier survival curves illustrate the primary and secondary outcomes (Figure 3).

**Table 3:** Follow‐up outcomes.

Use of AAD after ablation	49 (75.4%)
Amiodarone—*n*	38
Sotalol—*n*	6
Mexiletine—*n*	3
Propafenone—*n*	1
Amiodarone + Sotalol—*n*	1
No AAD after ablation	16 (24.6%)
Hospitalization duration (days)	7 (2−51)
Follow‐up duration	23 (0−93)
VT related ICD therapies	22 (33.85%)
Secondary endpoint	47 (72.3%)
All cause mortality	29 (44.62%)
Cardiac	22
Intrahospital	3
During follow‐up	19
Noncardiac	7
Hospitalization	40 (61.54%)
Stroke	1 (1.54%)
Redo procedures performed	18 (27.7%)
Endocardial ablation	18 (100%)
Additional epicardial ablation required	8 (44.4%)
Epicardial ablation also in index procedure	6 (33.3%)
Epicardial ablation only at redo	2 (11.1%)
Redo target location versus index
Same location	8 (44.4%)
Adjacent or anatomically similar region	7 (38.9%)
Different region	3 (16.7%)

### 3.4 Patients Grouped Into Ischemic CMP and Non‐Ischemic CMP

In the ischemic group, the median follow‐up duration was 18 months (0–88 months). VT‐related ICD therapies occurred in 7 patients (18.9%), while 22 patients (59.5%) died, and 25 patients (67.6%) experienced secondary outcomes. In the non‐ischemic group, the median follow‐up duration was 29 months (1–93 months), with VT‐related ICD therapies reported in 15 patients (53.6%), mortality in 7 patients (25.0%), and secondary outcomes in 22 patients (78.6%). The log‐rank test indicated a statistically significant difference in VT‐related ICD therapy‐free survival (*χ*² = 4.87, *p* = 0.027) and mortality‐free survival (*χ*² = 6.18, *p* = 0.013) between the ischemic and non‐ischemic groups. However, there was no significant difference in secondary outcome‐free survival between the groups (*χ*² = 0.02, *p* = 0.877) (Supporting Information S1: Figure 1).

Univariate analysis showed that the type of cardiomyopathy, hypertension and coronary artery disease, LVEF, pulmonary artery pressure, severity of mitral regurgitation, BNP level on admission, and previous ablation history were associated with mortality. However, multivariate analysis revealed that only the LVEF was associated with mortality. The results of the regression analysis are detailed in Table 4.

## 4 Discussion

This study's main findings are that VT ablation is an effective treatment for patients with ES. Most patients can be safely discharged following ablation. Despite a high rate of acute procedural success, these patients remain at significant risk for mortality and morbidity. Reduced LVEF was identified as the most important predictor of mortality during follow‐up.

ES is a critical presentation, and intrahospital mortality and morbidity risk are high. It requires an advanced multidisciplinary approach [3, 8]. Recent guidelines suggest catheter ablation in patients with ES refractory to AADs. Previous trials highlighted the effectiveness of catheter ablation in this patient group [1, 3]. Even though the acute procedural success of VT ablation is high, patients with ES remain at significant risk for VT recurrence and mortality. VT ablation in patients with ES often requires more prolonged and complex ablation procedures. In‐hospital mortality in our cohort was 4.6%, which aligns with rates reported in previous studies [4, 9, 10] ranging from 0.0% to 7.5%. Our findings highlight the efficacy of VT ablation in patients with ES. Similar to prior studies, acute procedural success was high, demonstrating that VT ablation is an effective intervention to stabilize and discharge patients with ES without significant morbidity. Vergara et al. [4] reported a 20.1% mortality rate in patients with ES following ablation at 12 months. Benali et al. [9] documented 19% mortality in the first year and 29% in the third year. Similarly, the mortality rate was 26.15% at 12 months in our cohort.

In our study, the mortality rate was 40% over a median of 23 months, and the median survival time was estimated at 51 months. The composite endpoint rate, including all‐cause death, VT recurrence, and hospitalization, was 72%, while the estimated median survival rate for secondary outcomes was 26 months. With a median follow‐up of 702 days, Huang et al.￼ reported mid‐term results for patients with ES after early catheter ablation therapy, revealing a 47% occurrence of a composite endpoint that includes death, heart transplant, VT storm recurrence, and VT‐related hospitalization.

One of the interesting findings from our study was that when patients were categorized into ischemic and non‐ischemic etiologies, the follow‐up outcomes showed a variety. Although VT‐related ICD therapies were significantly higher in the non‐ischemic group, mortality was more prevalent in the ischemic group. Previous studies reported that VT ablation outcomes were poorer in non‐ischemic groups compared to ischemic cardiomyopathy, especially during longer follow‐up periods [11]. Our findings were consistent with this knowledge. While VT recurrence following ablation was higher in our cohort, left‐sided heart failure predominating in ischemic VT may cause such a higher mortality rate despite lower VT recurrences after ablation. This is because we know that the ablation outcomes in non‐ischemic patients are strongly related to the specific subgroup of non‐ischemic etiology, and our non‐ischemic patient subgroups were heterogeneous [12].

Our study also provides insights into the subgroup of patients who required repeat ablation. Redo procedures were necessary in approximately one‐quarter of the cohort. While all redo procedures involved endocardial access, epicardial ablation was additionally required in nearly half of the cases. Interestingly, two of these patients had not received epicardial ablation during their initial procedure, indicating that substrate progression or incomplete initial ablation might necessitate epicardial intervention at follow‐up. Importantly, the anatomical locations of redo ablation were generally concordant with or in close proximity to the index ablation sites, suggesting that recurrent arrhythmia may emerge from adjacent or overlapping areas of arrhythmogenic substrate.

Patients with ES face a significantly higher risk of mortality and VT recurrence compared to those with non‐storm VT episodes. Previous studies have highlighted that ES is associated with a 2.5‐fold increased risk of all‐cause mortality compared to sporadic VT episodes. The findings from our cohort align with these reports, underscoring the critical prognostic implications of ES in patients with structural heart disease. Multivariate regression analysis in our cohort showed that the type of cardiomyopathy is not related to mortality. However, reduced LVEF was the only significant predictor of mortality in patients with ES. Moreover, mortality was higher in our ischemic group. The mean LVEF was 29% in ischemic patients and 43% in non‐ischemic patients. This difference between the groups can be attributed to the difference in LVEF. Therefore, more comprehensive therapies following VT ablation should be urgently planned for patients with reduced LVEF and ES. Notably, the high rate of mortality and morbidity observed in our cohort suggests that ES is not merely an isolated arrhythmic episode but a sentinel event that reflects the progression and severity of underlying structural heart disease.

Although univariate analysis identified several potential predictors of mortality, LVEF emerged as the only statistically significant predictor in multivariate analysis. Similarly, Benali et al. [9] also highlighted LVEF as a key determinant of mortality. This finding underscores that follow‐up mortality is predominantly influenced by the progression and severity of the underlying structural heart disease.

For clinicians, recognizing ES as a marker of disease progression is essential. Admission due to ES should prompt a comprehensive re‐evaluation of the patient′s structural heart disease and long‐term prognosis [5]. While not curative, VT ablation is a highly effective intervention to palliate the acute deterioration associated with ES. It offers additional time for optimizing medical therapy and considering advanced interventions. For instance, Margoli et al. [13] demonstrated that VT ablation in ES patients extended transplant‐free survival, suggesting that ablation can serve as a bridge to advanced therapies without directly altering long‐term mortality.

Looking ahead, integrating VT ablation with advanced heart failure treatments, such as ventricular assist devices or heart transplantation, could further enhance outcomes in this high‐risk group. Prospective studies are needed to explore the role of adjunctive therapies, optimize intervention timing, and investigate novel ablation techniques, like pulsed field ablation, which may offer safer and more durable results for patients with ES. By addressing the disease's arrhythmic and structural aspects, a multidisciplinary approach will remain essential for improving survival and quality of life in these patients.

This study contributes to the existing literature by providing long‐term outcome data on VT ablation in patients with ES. This population remains at high risk for mortality and morbidity despite successful acute interventions. While prior studies have established the efficacy of VT ablation in achieving acute procedural success, our findings underscore the persistent long‐term risks and the necessity for additional therapeutic strategies beyond ablation alone. By demonstrating that a significant proportion of patients experience recurrent VT events, hospitalizations, and mortality despite ablation, this study highlights the urgent need for multidisciplinary post‐ablation management approaches, including heart failure optimization and advanced heart failure therapies. Future research should focus on integrating ablation with novel adjunctive strategies, such as ventricular assist devices and emerging ablation technologies, to improve long‐term survival in this high‐risk cohort.

Our trial has several limitations. First, it employs a retrospective design. Because of the retrospective and observational design of this study, no formal power calculation was conducted before patient enrollment. The study included all eligible patients consecutively to reduce selection bias. Second, the cohort size is relatively small, and the patients' structural diseases are heterogeneous. Third, while the demand for heart transplantation is considerable in this specific population, none of our patients had the opportunity for transplantation, and 19 of the patients (29.2%) died due to cardiac disorders during follow‐up. Another limitation is the potential confounding effect of preprocedural AAD therapy on VT inducibility. AADs given before ablation, especially intravenous amiodarone due to its long half‐life, may suppress VT inducibility during electrophysiological testing. This pharmacologic suppression could potentially lead to an overestimation of procedural success rates. Another key limitation is the lack of a comparator group treated exclusively with antiarrhythmic drug therapy. Therefore, while our results indicate positive outcomes following VT ablation in ES, we cannot definitively evaluate the effectiveness of ablation compared to medical therapy alone. Although our results highlight the importance of additional treatment options alongside VT ablation, the efficacy of these therapies cannot be accurately assessed, and further studies are necessary.

## 5 Conclusion

VT ablation achieves high acute procedural success, enabling most patients to be discharged after the intervention. However, this high‐risk patient group continues to experience significant morbidity and mortality. While VT ablation provides a critical means to extend survival, complementary therapeutic strategies targeting the underlying structural heart disease are crucial to further reduce the long‐term risks of morbidity and mortality.

## Author Contributions

Conceptualization: Cem Çöteli and Kudret Aytemir. Methodology Development: Hikmet Yorgun. Data Acquisition: Samuray Zekeriyayev and Can Sezer. Data Analysis and Interpretation: Cem Çöteli and Can Sezer. Manuscript Drafting: Cem Çöteli and Samuray Zekeriyayev. Critical Revision: Hikmet Yorgun and Kudret Aytemir.

## Ethics Statement

This retrospective study was conducted in accordance with the Declaration of Helsinki and approved by the institutional ethics committee of Hacettepe University. Informed consent was obtained from all participants.

## Conflicts of Interest

The authors declare no conflicts of interest.

## Supporting information


**Figure 1a:** The Kaplan‐Meier survival curves demonstrate the VT related therapy free survival probabilities for ischemic and nonischemic groups. **Figure 1b:** The Kaplan‐Meier survival curves illustrate the all‐cause mortality free survival probabilities for the two groups. **Figure 1c:** The Kaplan‐Meier survival curves show the secondary outcome free survival probabilities for the two groups.

## Data Availability

The data sets used and analyzed during the current study are available from the corresponding author on reasonable request.
