# Supplementary material for: Long‐Term Outcomes of Catheter Ablation in Ventricular Tachycardia Electrical Storm: A Retrospective Cohort Study
Source: Clin Cardiol. 2025 Nov 15;48(11):e70221. doi: 10.1002/clc.70221 (PMC12619898; doi:10.1002/clc.70221)
Supplement: Supplementary file 1 — Figure 1a: The Kaplan‐Meier survival curves demonstrate the VT related therapy free survival probabilities for ischemic and nonischemic groups. Figure 1b: The Kaplan‐Meier survival curves illustrate the all‐cause mortality free survival probabilities for the two groups. Figure 1c: The Kaplan‐Meier survival curves show the secondary outcome free survival probabilities for the two groups. [file CLC-48-e70221-s001.docx]

| 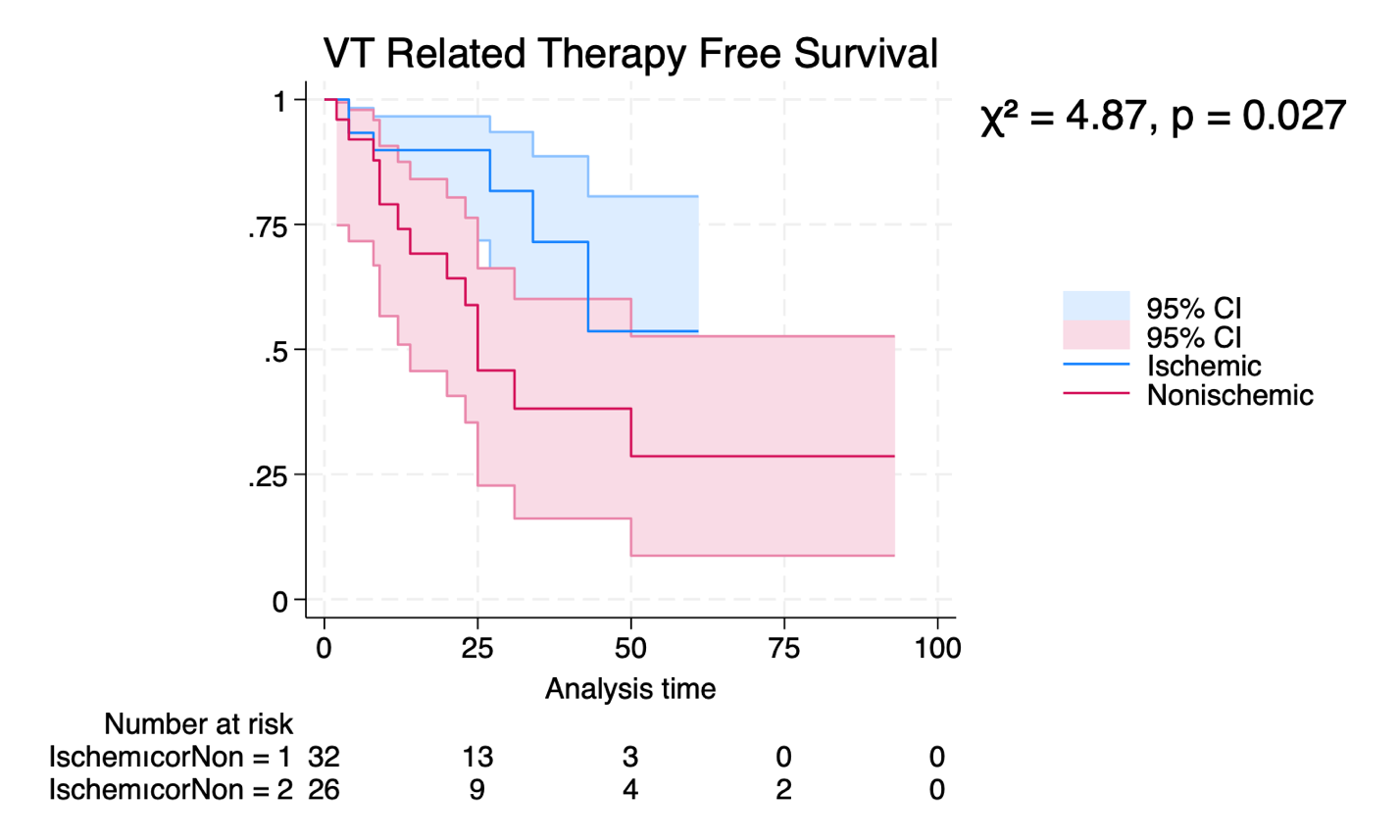 |
| --- |
| Supplementary Figure 1a. The Kaplan-Meier survival curves demonstrate the VT related therapy free survival probabilities for ischemic and nonischemic groups. The log-rank test revealed a statistically significant difference in VT related therapy free survival between the two groups (χ² = 4.87, p = 0.027). |
| 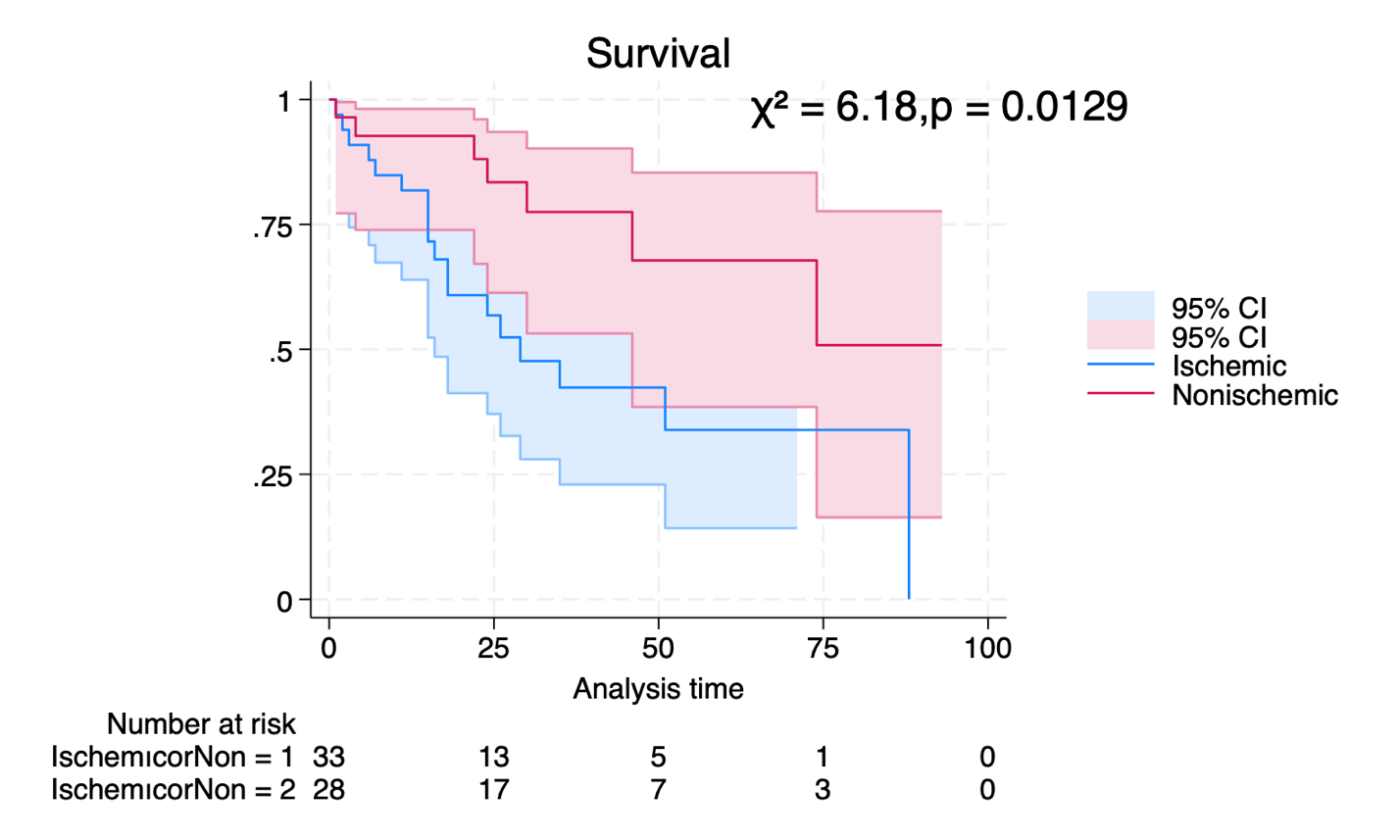 |
| Supplementary Figure 1b. The Kaplan-Meier survival curves illustrate the all-cause mortality free survival probabilities for the two groups. The log-rank test demonstrated a statistically significant difference in all cause mortality free survival between the groups (χ² = 6.18, p = 0.013). |
| 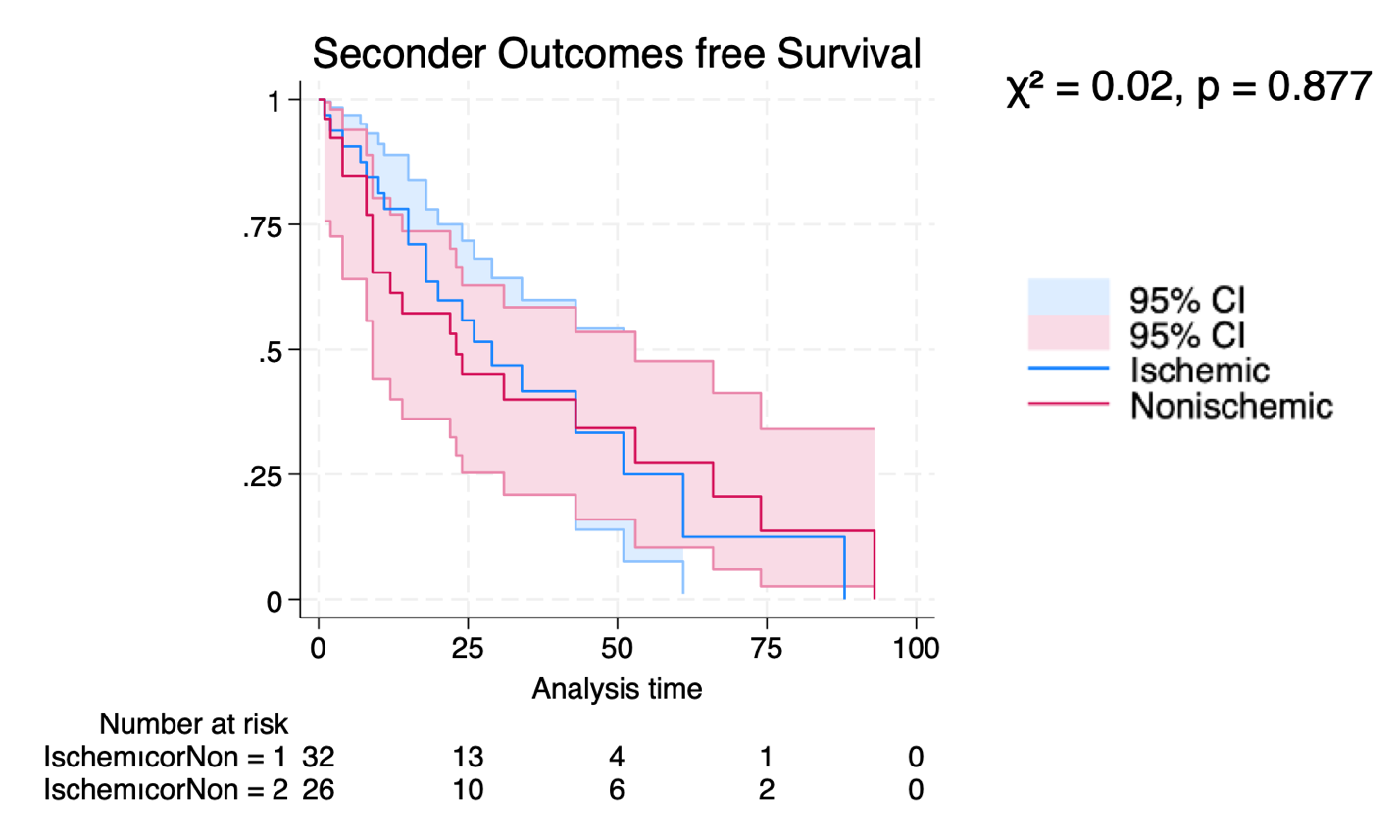 |
| Supplementary Figure 1c. The Kaplan-Meier survival curves show the secondary outcome free survival probabilities for the two groups. The log-rank test indicated no statistically significant difference in the secondary outcome free survival between the groups (χ²= 0.02, p = 0.877). |
